# Army and Navy Notes

**Published:** 1898-06

**Authors:** 


### ARMY AND NAVY NOTES.

The war department, at the instance of Surgeon-General Sternberg, has chartered the steamship John Englis, of the Maine Steamship Company, to be used for the purpose of a floating hospital to accompany the army when it undertakes the occupation of Cuba. The Vigiliancia, a comparatively new vessel of generous proportions, was first proposed, but upon inspection she was pronounced inadequate. The John Englis, therefore, has been selected. In addition to serving the purposes of a hospital ship the steamer will become a supply depot from which requisitions for medicines for the use of the troops in the field will be filled. She will also carry a large quantity of ice, which is especially needed in the tropics.

Surgeon-General Sternberg has appointed the following-named physicians who are immune from yellow fever, for service in the army: Dr. John Guiteras, of Philadelphia, who will be stationed at Tampa; Drs. W. E. Parker, and W. W. Calhoun, of New Orleans, also to be stationed at Tampa; Dr. Bernard E. Baker, of Charleston, who will be stationed at Key West, and Dr. Aristides A. Gramonte, a Cuban by birth and who was connected with the health department at New York city. The pay pertaining to these appointments is $150 a month.

Surgeon-General Sternberg is completing preparations for utilising the convent at Key West, which has been placed at his disposal by the sisters in charge for hospital purposes. Accommodations will be made for 250 beds. In addition the department, if necessary, will avail itself of several sugar factories at Key West where there are accommodations for 300 or more beds.

Upon the urgent recommendation of Surgeon-General Sternberg the number of new ambulances ordered under emergency requisition for immediate manufacture on May 3, 1898, was increased from 125 to 325, and the extra 200 have been distributed among the leading wagon builders of the West. These ambulances are to be ready, under severe penalty, within a month from the above date, a portion to accompany the forces in Cuba and the remainder to be distributed to the volunteer army corps encampments.

---

SURGEON G. H. TORNEY, U. S. army, of West Point, who was once an assistant surgeon in the navy, has been charged with the duty of fitting out the John Englis for her new uses. She will be equipped with hospital rooms, medicine chests, refrigerating plant and a huge ice box for carrying a large quantity of ice and keeping it in a hot climate. Major Torney will be chief surgeon and he will have five other medical men with him. There will also be six trained women nurses, who are to be secured from the Johns Hopkins hospital, at Baltimore. They will be hired for this work and not enlisted.

---

THE 65TH REGIMENT, N. G. N. Y., has entered the United States service for the war with Spain. It left Buffalo May 1, 1898, for the state camp at Hempstead, L. I., and was escorted to the Erie railway station by a large military and civic procession amidst the plaudits of more than 50,000 people. Its field and staff is composed as follows :

Colonel, S. M. Welch, Jr.; Lieutenant-Colonel, William H. Chapin; Regimental Adjutant, First Lieutenant Walter F. Nurzey; Regimental Quartermaster, Capt. G. Reed Wilson; Surgeon, Maj. A. H. Briggs; Assistant Surgeon, First Lieutenant Harry Mead; Assistant Surgeon, First Lieutenant E. L. Ruffner; Chaplain, The Rev. H. S. Fisher; Major, First Battalion, C. E. P. Babcock; Adjutant, First Battalion, First Lieutenant J. W. Scribner; Major, Second Battalion, E. A. Smith, M. D.; Adjutant, Second Battalion, First Lieutenant Frank M. Chapin; Major, Third Batallion, J. D. Howland, M. D.; Adjutant, Third Batallion, First Lieutenant A. S. Bixby.

The regiment was mustered into the United States service May 17, and proceeded to Falls Church, Va., May 19, 1898. As Buffalo's first contribution of troops for the war with Spain its progress will be watched with great interest by every patriotic citizen.

---

DR. LEONARD B. ALMY, of Norwich, Conn., says the Norwich *Courier*, has been tendered by the Surgeon-general of the U. S. army a commission as medical director of a division in the volunteer service with the rank of major. Dr. Almy is at present at Camp Haven, where he is performing duty as a member of the examining board.

THE war department has issued a general order giving extracts from the treaty articles of the Geneva convention bearing on the neutrality to be accorded equipments and individuals engaged in the treatment of the sick and wounded. The department also publishes the regulations which have been adopted for the observance of the treaty, including the requirement that on persons and equipment engaged in caring for the sick and wounded the red cross shall be displayed.

Article VI. of the convention says specifically that wounded or sick soldiers shall be entertained and taken care of, to whatever nation they belong. Those who are recognised after their wounds are healed as incapable of serving shall be sent back to their country. The others may also be sent back on condition of not again bearing arms during the continuance of the war. Commanders-in-chief have the power to deliver immediately to the outposts of the enemy soldiers who have been wounded in an engagement, when circumstances permit this to be done.

---

COMMANDER ANDREW DUNLAP has been placed in command of the Solace instead of Lieutenant McKethan as stated in the issue of this journal for May. A full description of this naval ambulance ship, the first to be commissioned by any government in the world, is given in the *Medical Record* May 14, 1898. It is written by past assistant-surgeon Edward S. Bogert, Jr., one of the medical officers of the Solace, and is illustrated by section drawings of the several decks and a half-tone photograph of the ship under full sail, flying the Red Cross flag at her fore.

---

THE Solace has already been of service in action, having received the wounded from the New York after the bombardment of San Juan de Porto Rico by Admiral Sampson, May 12, 1898. In case of an early meeting of the American fleet and the Spanish squadron in Cuban waters, as is anticipated at this writing, it is probable that the Solace will convey the wounded with all possible haste to New York instead of to Key West. In anticipation of the Solace coming to New York, where the hospital facilities are so much superior to those at ports farther south, elaborate arrangements have been made for receiving the wounded and caring for them. If Sampson is as fortunate as Dewey was in the matter of casualties, which can scarcely be possible, the Solace will not return to port, but will continue to cruise with the squadron.

The Adjutant General of the United States army recently advised Governor Hastings of the necessary physical examinations requisite before volunteers would be permitted to join a state encampment. The result is that an army surgeon and two civilian physicians will constitute such a board of examiners for the state of Pennsylvania. Governor Hastings has therefore appointed Dr. William Pepper, of Philadelphia, and Dr. W. S. Foster, of Pittsburg, to conduct such examination on the part of physicians. In this way it is hoped to restrain those who are physically disabled from joining the militia. Would it not be well to adopt a similar system in New York?

---

The President has appointed William Sturgis Thomas to be assistant-surgeon in the navy. Dr. Thomas resides at 68 W. 52d street, New York.

---

The following appointments of medical officers for duty in the field have been announced from Washington: Colonel Charles R. Greenleaf, assistant surgeon-general, chief surgeon of troops in the field, attached to the staff of Major-General Miles, commander-in-chief. To be chief surgeons, with rank of lieutenant-colonel, Majors Benjamin F. Pope, surgeon United States army; Robert M. O'Reilly, surgeon United States army; Alfred C. Girard, surgeon United States army; John Van R. Hoff, surgeon United States army; Louis M. Maus, surgeon United States army; Rush Huidekoper of Pennsylvania; Nicholas Senn, of Illinois. To be chief surgeons with the rank of major: William H. Daly, of Pittsburg, Pa.; Clayton Parkhill, of Denver, Col.; Herbert W. Cardwell, surgeon-general, of Oregon; James H. Hyssell, of Pomeroy, O.; Henry F. White, of St. Paul, Minn.; Jefferson D. Griffith, medical director National Guard of Missouri; R. Emmet Giffen, surgeon-general of New York; E. Bockmann, National Guard of Minnesota; Thomas S. Kimball, of Marion, Ind.; Charles B. Nancrede, professor of surgery, University of Michigan; John McG. Woodbury, of New York; Lewis Schooler, of Iowa; George Cook, of Concord, N. H.; James M. Jenne, surgeon-general of Vermont; Leonard B. Almy, medical director, N. G. of Connecticut; Thomas Earle Evans, of Woodward, Ala.

---

The selection of medical officers for the 65th N.Y. Volunteers reflects credit upon the profession of Buffalo. Dr. Albert H. Briggs, who has been appointed surgeon, has been an established practitioner of medicine since 1871, and is well and favorably known in and out of the profession as a well-equipped physician of excellent judgment and undoubted skill. Drs. Mead and Ruffner, who have been appointed assistant-surgeons, are young men of excellent professional standing and will doubtless prove faithful coadjutors of Surgeon Briggs in the medical and surgical care of the officers and men of the 65th, to whom the JOURNAL tenders its congratulations upon this significant fact.

THE 65th N. Y. Volunteers has, besides its regular medical staff, two battalion majors who are physicians. Dr. Eugene A. Smith, major of the second battalion, is one of Buffalo's best known physicians and a surgeon of great skill and judgment. Dr. John D. Howland, a graduate of Niagara university, '91, is major of the third battalion. Dr. Howland is a young man of promise in his profession and will doubtless make his mark as a field officer.

SURGEON-GENERAL STERNBERG of the army has become attending physician to the President and residents of the White House. Past assistant-surgeon Wood, U. S. army, formerly the president's physician, has been appointed colonel of the famous cavalry regiment which is rendezvousing at San Antonio, Texas. The President is foıtunate in the selection of his medical adviser.

THE organisation of a hospital corps for the army of invasion is, according to Washington dispatches of recent date, well under way and may be outlined as follows: It will be divided into regimental detachments, ambulance companies, field hospitals, lines of communication hospitals, base hospitals, hospital transports and railway trains, and general hospitals already established in the United States. The last two will be under the immediate direction of the surgeon-general in Washington, and the first five will be in charge of Colonel Greenleaf, assistant surgeon-general attached to the staff of General Miles. Men who are wounded in action will be cared for by those divisions in the order of their numbering, the slightly wounded and the sick being discharged from the field hospitals if their injuries or illnesses are slight and they are capable of quickly returning to duty. Those whose condition is more serious will be transported—carried through the lines of communication hospitals if necessary—to the base hospitals, and only the bad cases who are unfitted for further active service will be sent in the hospital transports and specially fitted railway trains to the general hospitals in the United States.

About 180 medical officers, 60 surgeons and 120 assistant surgeons, will accompany the invading army, the ratio being one medical officer to about 400 soldiers of the line. The medical service will also include about 160 hospital stewards and acting stewards and about 1,200 private soldiers. In addition to the usual equipment of stretchers, 150 ambulances of the most modern design will be used, 100 of which are already with the troops, and remaining 50 were ordered shipped from Indiana to Tampa a few days ago. Not more than one-third of the entire sanitary organisation will accompany the first force of regulars that lands in Cuba, the remainder following with the volunteers.

